# Education Modifies the Association of Wealth with Obesity in Women in Middle-Income but Not Low-Income Countries: An Interaction Study Using Seven National Datasets, 2005-2010

**DOI:** 10.1371/journal.pone.0090403

**Published:** 2014-03-07

**Authors:** Amina Aitsi-Selmi, Ruth Bell, Martin J. Shipley, Michael G. Marmot

**Affiliations:** Department of Epidemiology and Public Health, University College London, London, United Kingdom; Indiana University, United States of America

## Abstract

**Background:**

Education and wealth may have different associations with female obesity but this has not been investigated in detail outside high-income countries. This study examines the separate and inter-related associations of education and household wealth in relation to obesity in women in a representative sample of low- and middle-income countries (LMICs).

**Methods:**

The seven largest national surveys were selected from a list of Demographic and Health Surveys (DHS) ordered by decreasing sample size and resulted in a range of country income levels. These were nationally representative data of women aged 15–49 years collected in the period 2005–2010. The separate and joint effects, unadjusted and adjusted for age group, parity, and urban/rural residence using a multivariate logistic regression model are presented

**Results:**

In the four middle-income countries (Colombia, Peru, Jordan, and Egypt), an interaction was found between education and wealth on obesity (*P*-value for interaction <0.001). Among women with no/primary education the wealth effect was positive whereas in the group with higher education it was either absent or inverted (negative). In the poorer countries (India, Nigeria, Benin), there was no evidence of an interaction. Instead, the associations between each of education and wealth with obesity were independent and positive. There was a statistically significant difference between the average interaction estimates for the low-income and middle-income countries (P<0.001).

**Conclusions:**

The findings suggest that education may protect against the obesogenic effects of increased household wealth as countries develop. Further research could examine the factors explaining the country differences in education effects.

## Introduction

The 2011 UN High Level Summit gave global prominence to the growing problem of non-communicable diseases (NCDs), particularly heart disease and diabetes for which obesity is a key risk factor [1]. It is arguably the most complex to address and presents a potentially intractable epidemic in itself [2]. Cross-country comparisons have played a central role in understanding the association between socio-economic status (SES) and obesity in relation to country levels of economic development [3], [4], [5], [6]. Most of this research suggests that obesity is a problem of the elites in poorer countries, with a process of gradual reversal occurring as countries develop, and it becomes a problem of the poor in advanced economies.

Recent studies have disputed whether the reversal is taking place [7], [8], [9]. However, they favoured wealth as the better indicator and neglected the possibility that different SES indicators might act differently. Education has been shown to have a different association with mortality compared with occupation in high-income countries and a different association with heart disease compared with indicators of material circumstances in ex-Communist countries [10], [11].

In developing economies, rising income or wealth may be used to purchase greater quantities of food which increases obesity risk [12] and this effect may be exacerbated by a mismatch between a biology programmed in early life for scarcity and the exposure in later life to a sudden influx of cheap calories as markets open to the global food system [13]. On the other hand, education may mitigate this effect through cognitive skills that influence information processing and health-related decision-making [14], [15]. In other words, individuals may be able to modify their response to an increasingly obesogenic environment by drawing on personal resources such as educational capital and cognitive skills to navigate new disease risks. This may be useful where interventions targeting the food industry are politically unfeasible.

In a previous study [16], we tested the hypothesis that education and wealth may have separate but inter-related effects on obesity in a country with a high level of female obesity (Egypt). Having confirmed the existence of an interaction whereby education modified the positive wealth-obesity association, we then sought to examine whether this finding could be replicated in other middle-income countries. In addition, we aimed to test the hypothesis that the protective role of education was dependent on the level of economic development of a country in line with the reversal, such that this protective role would be found in middle-income but not low-income countries. In the latter, associations between any marker of SES and obesity might be positive. This is because the food environment is more likely to be dominated by food scarcity rather than obesogenic drivers including increased calorie availability and commercial marketing. As a result, there would be a minimal role for education to be protective against obesity in low-income countries, where any socioeconomic advantage might be used for calorie accumulation.

Therefore, this study examines the association between education and wealth in relation to obesity in women in a representative sample of low- and middle-income countries. It tests the hypothesis that education may protect against the association of wealth with obesity in middle-income countries and that this would not be observed in low-income countries. It aims to open up new avenues of investigation for the separate and inter-related effects of wealth and education on obesity in emerging economies.

## Methods

### Dataset

The Demographic and Health Surveys (available: DHS - http://www.measuredhs.com/Data/. Accessed 2013 Jan 1) are a key source of data for studies on obesity [4], [5], [6], [7], [8], [17], [18], [19]. They are a worldwide project funded by the United States Agency for International Development aiming to provide data on demographics and health outcomes, mainly for women and young children. They are nationally representative household-based, cross-sectional surveys using a multistage stratified probabilistic sampling design. The primary population surveyed is the universe of all ever-married women aged 15–49 years [20]. The standardisation of the methodology and survey instruments combined with extensive interviewer training make them ideal for cross-country comparisons.

### Study sample

All countries for which a DHS survey had been conducted between 2005 and 2010 (the latest wave at the time of this study) were eligible. They were listed according to sample size using the DHS STAT compiler tool available on the DHS website (available: http://www.statcompiler.com. Accessed 2013 Jan 1). We selected the six countries with the largest datasets for comparison with the Egyptian DHS dataset used in a previous study [16]. They included Benin, India, Nigeria, Peru, Jordan and Colombia and represented a range of country income levels based on the World Bank country income classification at the time of the survey (available: http://data.worldbank.org/about/country-classifications> and notes http://tinyurl.com/cnhf9aw. Accessed 2012 Nov 10). Jordan was selected to satisfy an additional sampling criterion of selecting another country from the Middle East and North Africa (MENA) region to provide a closer comparison with Egypt culturally. Table 1 shows the survey characteristics by country including response rates which were all above 90%. The DHS survey waves that were selected recorded anthropometry for all ever-married women (unlike earlier waves which took measurements from women with young children only). We excluded pregnant women (see Table 1), but we did not exclude extreme values as there were too few to influence the estimates and variance.

**Table 1:** Sample details by economic classification at the time of the DHS survey for each country included in the analysis.

	Benin	India	Nigeria	Egypt	Jordan	Peru	Colombia
GNIpc at time of survey ($)^1^	590	720	1170	1250/1880	3110	3590	5510
Income classification^2^	LI	LI	LI	MI	MI	MI	MI
DHS Year	2006	2005	2008	2005/08	2007	2008	2010
Number of women interviewed	17794	124385	33558	36045	10876	16159	53320^3^
Response rate of women eligible for interview	94	95	97	>99	98	98	94^3^
Women with complete anthropometry	16717	118734	32358	35690	5196^4^	15228	49637
Missing covariates (% of anthropometry sample)	0	<0.01%	0	0	0	0	7.6%
Final analytic sample^ 5(figures below relate to this sample)^	14883	113063	28901	32272	4527	14483	47709
							

1 GNIpc in $ using Atlas method at time of survey, World Bank classification. http://tinyurl.com/3bpg77q.

2 World Bank income classification. <http://data.worldbank.org/about/country-classifications> and notes http://tinyurl.com/cnhf9aw>. LI = low-income; MI = middle-income.

3 Includes women 13–49 years (the rest of the figures for Colombia are for women 15–49 years).

4 Anthropometry collected in half of the household sample.

5 Excludes pregnant women; includes missing covariates.

### Outcome and covariates

The model used in this study is based on the literature of multicountry studies examining SES-obesity association and includes a relatively small number of variables [4], [5], [7], [17], [19]. Body mass index (BMI in kg/m^2^) was calculated as (weight/height^2^) and the cut off for obesity was defined as a BMI≥30 kg/m^2^. Participants were weighed on a digital scale and their weight recorded in kilograms to the nearest 100 grams. Height was measured using an anthropometer with standard gradations and recorded to the nearest millimetre.

Education level was based on self-reported educational attainment recorded by the interviewers. It was coded into two categories (1 = no/primary education, 2 =  secondary/higher education) for the main part of the analysis to ensure sufficient numbers in the subgroups. A second analysis was conducted with a three-level education variable (1 = no/primary education, 2 =  secondary, 3 = higher) in the middle-income countries to add further differentiation to the education variable and examine dose-response. Age group in ten year bands and parity (number of children) were included as biological confounders [21], and urban/rural residence as a simple adjustment for environmental exposures.

Parity was used as a categorical variable with four groups (0; 1–3; 4–6; 7+). Parity is thought to be an important factor related to obesity in high income countries [22] and low-and-middle income countries (LMICs) [21]. Women with a greater number of children are more susceptible to excess weight due to a number of factors including physiological and psychological factors. LMICs have much higher fertility rates than HICs as well as greater variation in the number of children between women of different social groups and, therefore, parity is likely to have even greater importance as a biological confounder [21].

The wealth index is generated for each country separately and places individuals in relative position to each other on a locally appropriate continuous scale of economic status [23]. It was originally developed to measure the ability to pay for health services but is also used as a general indicator of material circumstances [24]. Data on the ownership of durable assets such as electrical equipment (e.g. TV, computer), basic amenities (e.g. sanitation, water supply) and housing characteristics (e.g. floor material) are used to generate a wealth index score through principal components analysis based on the Filmer & Pritchett method [23]. This is a data reduction technique where the correlations between the full set of asset variables are used to generate a number of uncorrelated principal components. Only the first principal component - the one that explains most of the variance of the indicator variables - is selected to derive a score for each household. It was divided into quintiles (1 = poorest; 5 = richest) for each country.

### Statistical analysis

Each country was analysed separately. Stata 12 SE® complex survey procedures were used with the survey weights provided with each dataset to account for the design effect and obtain nationally representative prevalence estimates. To test the hypothesis that education level modified the association between wealth and obesity, an interaction between education and wealth was fitted in the logistic regression models. Education was used as a categorical variable. We tested whether the effect of wealth across the quintiles could be adequately described using a single linear term by comparing models with wealth fitted as a categorical or linear term. This showed that the associations between wealth and obesity were adequately described using wealth as a linear term. In addition, to check this linearity assumption visually, we plotted the associations, using the log odds ratios, between wealth, education and obesity using wealth as a categorical variable.

In order to assess for multi-collinearity between the independent variables in the model, variance inflation factors were calculated [25]. The averages of the interaction estimates for the low- and middle-income countries were compared using formal statistical testing.

Four models are presented. The first two models provide estimates for the association between each of education and wealth with obesity separately. The estimates are presented both unadjusted and adjusted for age group, urban/rural residence and parity). The third model includes both education and wealth. The fourth model includes education, wealth and an interaction between education and wealth. The effect of wealth (the effect of an increase in one wealth quintile on the odds of obesity) in the no/primary education group is estimated from this model. Then, using the interaction estimate for education-by-wealth, we calculated the effect of wealth within the higher education groups. Likelihood ratio tests comparing the goodness of fit between the third and fourth models allowed the interaction effect between education and wealth to be tested.

### Ethical review

Demographic and Health Surveys (DHS) data collection procedures were approved by the Measure DHS (Demographic and Health Surveys) (Calverton, MC) Institutional Review Board and by the national body that approves research studies on human studies in each country. Written consent was obtained by the interviewers from each participant. The use of the DHS data for this particular study was approved by Measure DHS, and considered exempt from full review by University College London because the study is based on an anonymous, public-use data set with no identifiable information on the survey participants.

## Results

Table 1 shows details of the seven countries. Benin, India, Nigeria were low-income (LI) countries, while Egypt, Colombia, Jordan, Peru were middle-income (MI).

### Socio-demographic characteristics

Table 2 shows that the middle-income countries had higher levels of education and more urban populations. The obesity prevalence was strikingly high in Egypt and Jordan (43.6% and 30.5% respectively) and was more than double that of Peru and Colombia (14.1% and 14.3% respectively) which are richer countries.

**Table 2:** Participant sociodemographic characteristic in each country, DHS data.

	Benin		India		Nigeria		Egypt		Jordan		Peru		Colombia	
	14883		113063		28901		32272		4527		14483		47709	
	Total N	% (SE^1^)	Total N	% (SE^1^)	Total N	% (SE^1^)	Total N	% (SE^1^)	Total N	% (SE^1^)	Total N	% (SE^1^)	Total N	% (SE^1^)
Income bracket	LI		LI		LI		MI		MI		MI		MI	
BMI (kg/m^2^)														
Non-obese	14090	94.3 (0.3)	109075	97.2 (0.1)	27349	94.0 (0.3)	19146	57.0 (0.6)	2992	69.5 (1.2)	12307	85.7 (0.5)	40573	85.9 (0.2)
Obese BMI	793	5.7 (0.3)	3988	2.8 (0.1)	1552	6.0 (0.3)	13126	43.0 (0.6)	1535	30.5 (1.2)	2176	14.3 (0.5)	7136	14.1 (0.2)
Education														
None/primary	12463	82.9 (0.8)	52274	55.2 (0.6)	16810	54.2 (1.3)	16250	49.1 (0.7)	793	12.7 (0.9)	4462	28.1 (1.3)	13489	24.4 (0.4)
Secondary/higher	2420	17.1 (0.8)	60789	44.8 (0.6)	12091	45.8 (1.3)	16022	50.9 (0.7)	3734	87.3 (0.9)	10021	71.9 (1.3)	34220	75.6 (0.4)
Urban/rural residence														
Urban	6407	42.6 (2.1)	51030	32.4 (1.0)	9256	36.4 (1.8)	13349	41.6 (1.5)	3102	84.2 (1.8)	9919	72.9 (2.1)	34210	77.5 (0.8)
Rural	8476	57.4 (2.1)	62033	67.6 (1.0)	19645	63.6 (1.8)	18923	58.4 (1.5)	1425	15.8 (1.8)	4564	27.1 (2.1)	13499	22.5 (0.8)
Age group (years)														
15–24	5077	34.6 (0.5)	41156	36.7 (0.2)	10995	37.9 (0.4)	5361	16.3 (0.3)	506	11.8 (0.7)	4903	33.5 (0.5)	14827	30.7 (0.3)
25–34	5175	34.7 (0.5)	34726	30.6 (0.2)	8930	31.1 (0.3)	11414	35.4 (0.3)	1726	37.0 (1.1)	4091	28.5 (0.6)	12259	25.6 (0.3)
35–49	4631	30.7 (0.4)	37181	32.7 (0.2)	8976	31.0 (0.4)	15497	48.2 (0.4)	2295	51.2 (1.2)	5489	38.0 (0.6)	16864	36.3 (0.3)
Children born														
0	3335	22.9 (0.5)	35590	28.1 (0.2)	8550	30.6 (0.6)	2388	7.1 (0.2)	307	6.4 (0.6)	4466	33.0 (0.7)	16134	35.1 (0.3)
1–3	5215	35.3 (0.5)	52839	47.1 (0.3)	8774	30.3 (0.4)	17759	57.2 (0.5)	1693	40.5 (1.1)	6945	47.9 (0.7)	24409	52.6 (0.3)
4–6	4055	27.1 (0.4)	20577	20.3 (0.2)	6821	23.4 (0.3)	9386	28.4 (0.3)	1636	37.6 (1.0)	2351	14.9 (0.5)	6010	10.7 (0.2)
7+	2278	14.7 (0.4)	4057	4.6 (0.1)	4756	15.6 (0.4)	2739	7.2 (0.2)	891	15.5 (0.9)	721	4.2 (0.3)	1156	1.6 (0.1)
**Wealth by education level**														
None/primary														
Poorer 40%	5334	51.9 (1.6)	23277	70.8 (0.8)	10240	73.0 (1.5)	10338	75.7 (1.0)	563	80.2 (3.0)	2590	74.9 (2.4)	10001	79.0 (0.9)
Richer 40%	4510	48.1 (1.6)	15980	29.2 (0.8)	3108	27.0 (1.5)	2753	24.3 (1.0)	82	19.8 (3.0)	711	25.1 (2.4)	1448	21.0 (0.9)
Secondary/higher														
Poorer 40%	153	6.1 (0.7)	5344	16.5 (0.6)	1661	12.7 (1.0)	3032	21.1 (0.9)	1791	48.6 (2.7)	1388	13.9 (1.3)	14421	46.7 (1.0)
Richer 40%	2044	93.9 (0.7)	46694	83.5 (0.6)	8190	87.3 (1.0)	9850	78.9 (0.9)	1111	51.4 (2.7)	5970	86.1 (1.3)	12102	53.3 (1.0)

1 SE  =  standard error of the estimated proportion x 100.

### Obesity prevalence

Table 3 shows that the prevalence of obesity was higher in the wealthier and older women, and tended to be higher in women with a greater number of children. It increased with education in the poorer countries but decreased with education in the richer countries. The absolute difference in prevalence between the richest and the poorest wealth groups diminished with increasing education level in the middle-income countries.

**Table 3:** Prevalence of obesity by sociodemographic characteristic in each country, DHS data.

	Benin		India		Nigeria		Egypt		Jordan		Peru		Colombia	
	N = 14883		N = 113063		N = 28901		N = 32272		N = 4527		N = 14483		N = 47709	
	N obese	% (SE^1^)	N obese	% (SE^1^)	N obese	% (SE^1^)	N obese	% (SE^1^)	N obese	% (SE^1^)	N obese	% (SE^1^)	N obese	% (SE^1^)
Income bracket	LI		LI		LI		MI		MI		MI		MI	
Education														
None/primary	567	4.9 (0.3)	1194	1.7 (0.1)	679	4.4 (0.2)	6682	44.4 (0.7)	355	47.3 (3.3)	799	17.2 (1.1)	2910	21.9 (0.5)
Secondary/higher	226	9.8 (0.8)	2794	4.2 (0.2)	873	7.9 (0.4)	6444	41.7 (0.7)	1180	28.0 (1.2)	1377	13.2 (0.5)	4226	11.6 (0.2)
Wealth quintile														
Poorest 20%	30	1.1 (0.2)	31	0.2 (0.0)	104	1.8 (0.2)	1938	30.6 (0.8)	415	30.4 (1.9)	49	4.3 (0.7)	1712	14.0 (0.5)
Poorer 20%	41	1.4 (0.2)	73	0.5 (0.1)	134	2.3 (0.2)	2285	37.6 (0.9)	344	27.7 (2.1)	323	9.4 (0.7)	2095	16.1 (0.5)
Middle 20%	89	3.2 (0.4)	219	0.9 (0.1)	235	4.1 (0.3)	2654	44.5 (0.9)	362	31.4 (2.7)	626	15.5 (0.9)	1497	14.6 (0.4)
Richer 20%	183	5.9 (0.4)	823	2.9 (0.1)	393	6.8 (0.4)	2988	49.2 (1.0)	271	34.7 (2.4)	570	18.0 (1.0)	1101	13.9 (0.5)
Richest 20%	450	13.7 (0.7)	2842	8.4 (0.3)	686	12.8 (0.6)	3261	51.4 (1.1)	143	28.4 (4.1)	608	15.6 (0.9)	731	11.9 (0.5)
Urban/rural residence														
Urban	570	9.7 (0.6)	3083	6.1 (0.2)	831	9.5 (0.5)	6416	49.5 (0.8)	1044	29.4 (1.4)	1691	16.2 (0.6)	5048	13.9 (0.2)
Rural	223	2.7 (0.2)	905	1.2 (0.1)	721	3.9 (0.2)	6710	38.4 (0.8)	491	36.3 (1.9)	485	9.2 (0.7)	2088	14.9 (0.5)
Age group														
15–24	85	1.8 (0.2)	281	0.5 (0.0)	190	1.7 (0.1)	895	17.8 (0.7)	60	11.8 (2.2)	205	3.9 (0.4)	883	5.3 (0.2)
25–34	265	5.5 (0.4)	1071	2.5 (0.1)	516	6.5 (0.4)	3775	34.8 (0.7)	411	21.2 (1.5)	606	13.8 (0.9)	1985	14.7 (0.4)
35–49	443	10.3 (0.6)	2636	5.7 (0.2)	846	10.6 (0.5)	8456	57.6 (0.7)	1064	41.5 (1.8)	1365	23.9 (1.1)	4268	23.9 (0.4)
Children born														
0	83	2.8 (0.4)	444	1.0 (0.1)	239	2.8 (0.2)	653	29.1 (1.2)	72	17.8 (3.4)	201	4.2 (0.5)	856	5.5 (0.2)
1–3	304	6.3 (0.4)	2676	4.0 (0.2)	473	6.4 (0.4)	6610	39.4 (0.7)	385	21.6 (1.6)	1255	17.6 (0.8)	4387	17.0 (0.3)
4–6	287	7.7 (0.6)	749	2.7 (0.2)	513	8.7 (0.5)	4594	52.0 (0.8)	619	33.0 (1.9)	571	23.7 (1.5)	1597	26.7 (0.8)
7+	119	5.2 (0.5)	119	2.1 (0.3)	327	7.3 (0.5)	1269	50.1 (1.2)	459	52.8 (2.8)	149	22.7 (2.7)	296	26.9 (1.8)
**Wealth by education level**														
None/primary														
Poorer 40%	69	1.3 (0.2)	90	0.4 (0.0)	213	2.1 (0.2)	3491	36.8 (0.8)	226	42.3 (3.9)	270	8.7 (0.7)	2027	20.6 (0.6)
Richer 40%	415	9.6 (0.5)	944	5.5 (0.3)	316	10.5 (0.6)	1651	60.4 (1.2)	48	56.6 (9.3)	243	34.9 (3.3)	365	24.0 (1.3)
Secondary/higher														
Poorer 40%	2	1.2 (0.9)	14	0.3 (0.1)	25	1.6 (0.4)	732	25.4 (1.0)	533	25.8 (1.3)	102	6.9 (0.9)	1780	10.8 (1.1)
Richer 40%	218	11.1 (0.8)	2721	5.8 (0.2)	763	9.7 (0.5)	4598	47.3 (0.9)	366	30.1 (2.4)	935	14.5 (0.7)	1467	12.1 (1.1)

1 SE  =  standard error of the estimated proportion x 100.

### Separate, independent and interaction effects of education and wealth by level of economic development

The variance inflation factors for the independent variables used in the model were less than three in all countries indicating that multi-collinearity was not a concern for the analysis. The wealth by education interaction estimates from the logistic regression models were heterogeneous across all seven countries and, in particular, the difference in the average of the interaction estimates in the low-income countries compared with the middle-income countries had a *P*-value of <0.001, thus, providing strong evidence of a difference between these two groups which are described separately in Tables 4 and 5 respectively.

**Table 4:** Separate, independent and interaction effects of education and wealth on obesity in the low-income countries, DHS data.

	Benin		India		Nigeria	
	N = 14883		N = 113063		N = 28901	
	OR (95%CI)		OR (95%CI)		OR (95%CI)	
	Unadjusted	Adjusted^1^	Unadjusted	Adjusted^1^	Unadjusted	Adjusted^1^
**Model using education**						
Education (level)						
None/primary	1	1	1	1	1	1
Secondary/higher	2.16 (1.84–2.53)	2.01 (1.67–2.44)	2.06 (1.92–2.21)	2.23 (2.07-2.41)	1.84 (1.68–2.04)	2.39 (2.11-2.70)
Model using wealth						
Wealth quintile (linear)	2.09 (1.94–2.23)	1.99 (1.84–2.16)	2.77 (2.65–2.89)	2.41 (2.30–2.53)	1.76 (1.68–1.83)	1.80 (1.70-1.90)
**Model both education and wealth**						
Education (level)						
None/primary		1		1		1
Secondary/higher		1.26 (1.04–2.12)		1.18 (1.09–1.28)		1.32 (1.16–1.52)
						
Wealth quintile (linear)		1.95 (1.79–2.12)		2.33 (2.23–2.46)		1.71 (1.62-1.81)
**Model including education and wealth and a wealth-by-education interaction** 2						
**Wealth effect within education level** 3						
None/primary		1.98 (1.81–2.15)		2.25 (2.11–2.39)		1.72 (1.61–1.84)
Secondary/higher		1.67 (1.30–2.16)		2.48 (2.29–2.70)		1.70 (1.55–1.86)
Interaction estimate^4^		0.85 (0.65– 1.10)		1.10 (1.00–1.21)		0.99 (0.89–1.10)
*P*-value for the interaction estimate^5^		0.2		0.06		0.8

1 Estimates adjusted for age group, urban/rural residence, and parity.

2 Estimates for education not shown in this section of the table.

3 OR for obesity associated with an increase in one wealth quintile within each education level calculated using the interaction estimate obtained from the model including an interaction between education and wealth.

4 Wealth effect in the secondary/higher education divided by the wealth effect in the no/primary education group e.g. for Benin, 0.85 = 1.67/1.98).

5 Test of whether the wealth effects differ by education level.

**Table 5:** Separate, independent and interaction effects of education and wealth on obesity in the middle-income countries, DHS data.

	Egypt		Jordan		Peru			Colombia	
	N = 32272		N = 4527		N = 14483			N = 47709	
	OR (95%CI)		OR (95%CI)		OR (95%CI)			OR (95%CI)	
	Unadjusted	Adjusted^1^	Unadjusted	Adjusted^1^	Unadjusted	Adjusted^1^		Unadjusted	Adjusted^1^
**Model using education**									
Education level									
None/primary	1	1	1	1	1	1		1	1
Secondary/higher	0.96 (0.92–1.00)	1.15 (1.09–1.22)	0.57 (0.49–0.67)	0.98 (0.85–1.12)	0.73 (0.66–0.80)	0.97 (0.86–1.09)		0.51 (0.49–0.54)	0.79 (0.74–0.84)
Model using wealth									
Wealth quintile (linear)	1.28 (1.26–1.30)	1.26 (1.23–1.29)	1.02 (0.98–1.07)	0.95 (0.90–0.99)	1.23 (1.18–1.27)	1.21 (1.15–1.28)		0.96 (0.94–0.98)	0.94 (0.91–0.96)
**Model using both education and wealth**									
Education (level)									
None/primary		1		1		1			1
Secondary/higher		0.86 (0.81–0.92)		0.86 (0.72–1.02)		0.84 (0.74–0.95)			0.81 (0.76–0.86)
								
Wealth quintile (linear)		1.30 (1.27–1.33)		0.96 (0.91–1.00)		1.24 (1.17–1.31)			0.96 (0.93–0.98)
**Model including education, wealth and a wealth-by-education interaction** 2									
**Wealth effect within education level** 3									
None/primary		1.39 (1.35–1.43)		1.27 (1.11–1.44)		1.65 (1.52–1.80)			1.09 (1.04–1.13)
Secondary/higher		1.19 (1.16–1.23)		0.91 (0.86–0.96)		1.08 (1.02–1.15)			0.92 (0.89–.94)
Interaction estimate^4^		0.85 (0.82–0.89)		0.72 (0.62–0.83)		0.66 (0.60–0.72)			0.84 (0.81–0.88)
*P*-value for the interaction estimate^5^		<0.001		<0.001		<0.001			<0.001

1 Estimates adjusted for age group, urban/rural residence, and parity.

2 Estimates for education not shown in this section of the table.

3 OR for obesity associated with an increase in one wealth quintile within each education level calculated using the interaction estimate obtained from the model including an interaction between education and wealth.

4 Wealth effect in the secondary/higher education divided by the wealth effect in the no/primary education group e.g. for Egypt, 0.86 = 1.19/1.39).

5 Test of whether the wealth effects differ by education level.

#### i.) Low-income countries (Benin, India, and Nigeria)

The top part of Table 4 shows a positive association between each of education and wealth before and after adjustment. In India (the largest sample), the unadjusted odds of obesity are 2.06 times higher (95%CI: 1.92, 2.21) for the secondary/higher education group *vs.* the group with no/primary education; and 2.23 times higher (95%CI: 2.07, 2.41) after adjustment. In terms of wealth, the odds of being obese increased by 2.77 times for each increase of one wealth quintile (95%CI: 2.65, 2.89) and remained positive after adjustment (OR; 95%CI: 2.41; 2.30, 2.53). A similar pattern was observed in Nigeria and Benin.

The middle part of Table 4 shows the independent effects of education and wealth from a model that includes both of these variables. The effect of education was much smaller in magnitude compared with the first part of the table, but the effect of wealth was little affected. This suggests that wealth may largely account for the apparent positive association between education and obesity observed in the low-income countries.

The bottom part of Table 4 illustrates the absence of evidence of an interaction (P>0.05), although the *P-*value for India (*P* = 0.06) was relatively closer to statistical significance than Benin or Nigeria (*P* = 0.2 and 0.8 respectively).

#### ii.) Middle-income countries (Egypt, Colombia, Jordan, and Peru)

In contrast to the low-income countries, the associations shown in Table 5 between education and obesity in the middle-income countries tended to be negative (OR<1) both before and after adjustment. In Colombia (the largest sample), the adjusted OR for the secondary/higher education group compared with the group with no/primary education was 0.79 (95%CI: 0.74–0.84). There was an inverse wealth-obesity association (adjusted OR; 95%CI: 0.94; 0.91, 0.96).

The middle part of Table 5 shows the independent effects of education and wealth in a model that includes both with the control variables. In contrast to the low-income countries, the association between education and obesity was inverse (<1). In Colombia, the association between wealth and obesity was negative (adjusted OR; 95%CI: 0.96; 0.93, 0.98).

**Figure 1:**
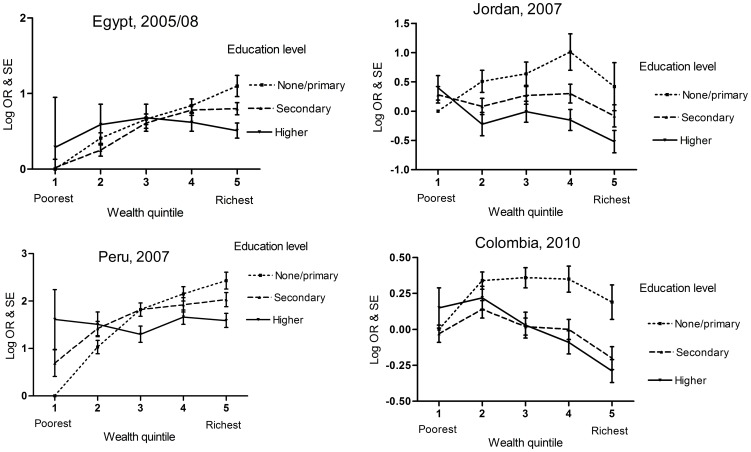
Interaction between education and wealth on the odds of obesity in women in middle-income countries. Each point represents the log OR of that combination of education level and wealth quintile compared with the reference category (education level = none/primary and wealth quintile = poorest). Error bars represent the standard error of the log OR. All plotted estimates are adjusted for age group urban/rural residence and parity.

The results for the regression model including the interaction between education and wealth are shown in the lower part of Table 5 as well as Figure 1. The comparison of models with wealth fitted as a categorical or linear term showed that the associations between wealth and obesity were adequately described as a linear term. Figure 1 illustrates these interactions using the log of the adjusted odds ratios and plots the associations between wealth, education and obesity using wealth as a categorical variable. The plots supported the use of wealth as a linear term. There was very strong evidence of an interaction for all countries (*P*<0.001). In the lowest education group, there was a positive wealth effect in all four countries. However, in Colombia and Jordan, the wealth effect was negative in the secondary/higher education group (OR; 95%CI: 0.92; 0.89, 0.94 and 0.91; 0.86, 0.96 respectively), while in Egypt and Peru the wealth effect in this group was non-significant. Of note, in both Jordan and Colombia - the countries with the strongest interaction effect - there is a negative association between wealth and obesity in the model with wealth alone and when wealth and education are mutually adjusted for (see Tables 5 and 6) rather than a positive association. For the countries where we were able to split education into three levels, as shown in Table 6, the results show that the positive wealth effect diminished progressively with higher levels of education.

**Table 6:** Effect of wealth on obesity within three education levels in the middle-income countries, DHS data.

	Egypt	Jordan	Peru	Colombia
	N = 32272	N = 4527	N = 14483	N = 47709
	OR (95%CI)^1^^,^ 2	OR (95%CI)^1^^,^ 2	OR (95%CI)^1^^,^ 2	OR (95%CI)^1^^,^ 2
**Wealth effect within education level** 3				
None/primary	1.39 (1.35–1.43)	1.27 (1.11–1.45)	1.65 (1.51–1.80)	1.09 (1.04–1.14)
Secondary	1.25 (1.21–1.29)	0.97 (0.90–1.04)	1.15 (1.07–1.25)	0.94 (0.91–0.98)
Higher	1.02 (0.93–1.13)	0.84 (0.76–0.93)	1.10 (0.98–1.23)	0.86 (0.82–0.91)
*P*-value for interaction^4^	*<0.001*	*<0.001*	*<0.001*	*<0.0001*

1 Model  =  education (three levels)*wealth quintile (continuous) + age group + urban/rural residence + parity.

2 ORs represent the effect of an increase in one wealth quintile on the odds of obesity within each education level.

3 Estimates for education not shown.

4 Test of whether the wealth effects differ by education level.

## Discussion

The objective of this study was to investigate the SES-obesity association in LMICs using two indicators of SES and to test the hypothesis that education may modify the association between wealth and obesity in middle- but not low-income countries. The findings show that while wealth tended to increase the odds of obesity in all countries, education appeared to have a protective role against the wealth effect in the middle-income countries (Egypt, Jordan, Peru and Colombia) only. Another finding was that the highest obesity rates were not in the richest countries.

### Comparison with prior studies

Literature reviews of the SES-obesity association have reported different associations by SES indicator including education and wealth [6], [26] and a number of single country studies from LMICs have reported separate effects of education and indicators of income or wealth including Peru, the Philippines, China and Brazil [27], [28], [29], [30]. No empirical studies have focused on investigating the relationship between different indicators or how these associations diverge by level of economic development for different indicators, although in a previous study, we have demonstrated an interaction between education and occupation in older Chinese women [31].

The findings in this study corroborate the existence of a changing SES-obesity association dependent on a country's level of economic development [3], [4], [5], [6]. However, two studies claim that DHS data do not provide any evidence of an SES-overweight/obesity reversal [7], [8]. This may be due to the use of a different outcome (overweight rather than obesity) or the use of fewer middle-income countries compared with a similar study supporting the reversal [32]. Notably, the authors place little emphasis on the education variable in their interpretation of the findings and focus on the wealth indicator which could mask the complexity of the social transition experienced in LMICs. Our findings concur with others emphasising that the social gradient of obesity is positive (high SES-high obesity) in India specifically [9], but also support the more general notion that obesity is a growing problem among those with lower education in LMICs. We propose a reinterpretation of the apparently contradictory studies [7], [8] and suggest that the education-obesity association may reverse before the wealth-obesity association.

### Plausible and competing mechanisms

There is a generally positive correlation between level of economic development and obesity levels with key correlates being urbanisation, calorie abundance, and women's participation in the labour market [2]. However, urbanisation appears to be less important than previously thought and a rapid rise in obesity levels among rural populations has been reported in middle-income countries recently [33], [34]. This is reflected in the differences in obesity prevalence by urban and rural residence in this study: the obesity prevalence is higher in both urban and rural areas in middle-income countries compared with low-income countries and the relative difference between the two areas is smaller. Data from China show that the prevalence of obesity has increased at a faster rate in the poorer rural areas than in the richer urban ones [35] and that lower income groups have disproportionately increased their consumption of animal fat and edible oil and reduced their consumption of healthier traditional foods [36].

One explanation is the increasingly widespread availability of processed, high-calorie food and drinks with such extensive supply chains that they are used for the delivery of antiretrovirals in countries where public health infrastructure is inadequate [37]. Aggressive marketing and strategic pricing in emerging economies are an important source of revenue for transnational corporations whose profits margins are decreasing in the West [38], [39], and new consumers may require greater cognitive resources to navigate these sophisticated economic signals. Therefore, as populations are increasingly exposed to processed, energy-dense foods, we can expect a general rise in obesity across all socioeconomic groups in middle-income countries, but especially among lower educated urban women.

The combination of persistent food insecurity in certain geographical areas (rural) and increased calorie availability overall may exacerbate the effects of the nutrition transition. In environments with longstanding food insecurity, metabolic programming and cultural memes transmitted between generations may favour the accumulation of calories [13], thus explaining the positive association between wealth and obesity. A rapid integration within the global economy resulting in a sudden influx of high-calorie products may alleviate the drive for calories before public health systems or health knowledge related to non-communicable diseases have taken root. This may result in a large information asymmetry between buyers and sellers [40]. Within this environment, having a higher level of education may provide an advantage through cognitive skills that can make up for the information asymmetry. Knowledge acquisition and information processing skills may assist in correcting cognitive biases created through marketing, thus resulting in clearer risk perception. In other words, education may extend capability, defined as a freedom that expands the range of options a person has in deciding what kind of life to lead, as well as developing judgment in relation to the appropriate exercise of choice [41].

There are a number of other possible pathways for the role of education which have been extensively discussed elsewhere [42], [43]. These include social norms and values since educated women may favour Western norms of thinness as a beauty criterion and use it as a means of social distinction [44]. These norms may be spread or reinforced within the educated elites through social network effects including through educated occupational classes which we could not control for in our analysis [45]. Educated women may come from different family backgrounds and their own mothers may have benefited from better education or food security thus reducing the risk of a metabolic mismatch between early and later life [46]. Finally, there is a link between marital status and obesity in high-income countries and educated women may be more likely to be unmarried. However, marital status did not explain the SES-obesity association in women in other LMICs [47].

### Strengths and limitations

The DHS datasets benefit from a highly standardised survey methodology and the results were remarkably consistent within country income groups. The cross-sectional nature of the data limits temporal and causal inferences, although this investigation did not seek to provide a causal analysis but to examine differences in patterns of association by level of economic development. Further research could investigate causal explanations for the country differences and/or possible pathways of explanation for the inverse education-obesity pathways in middle-income countries i.e. why are there opposite associations between education and wealth/income and obesity? Education may measure different things in different countries and women's occupation, marital status, body shape preference and husband's SES may have a greater or lesser importance depending on the country context.

It is also possible that reverse causality and health selection may be operating and that obesity determines educational and wealth opportunities. The pathways in this sample of countries are likely to be complex and vary from country to country. In Colombia, it is possible that being thin confers a social advantage while being obese is stigmatised, while the opposite might be the case in a low-income country like Benin. These cultural preferences are likely to vary by region (urban/rural) and by social group so that a more detailed country analysis and longitudinal data would be required to disentangle the direction of causality. However, this study aimed to engage with the current literature on the reversal of the SES-obesity gradient in women and makes a step forward in elucidating the complex social epidemiology of obesity in transition settings.

Finally, women under 17 years will not have had the opportunity to enter university and achieve the highest level of education, thus leading to the possibility of misclassification of education level. However, the number of women in this group was relatively small and is unlikely to change the patterns observed in these relatively large country samples.

### Conclusion

This study sought to separate the effects of women's education and wealth in relation to obesity. The findings support other studies documenting different SES-obesity associations and that the SES-obesity gradient reverses by level of economic development. The findings also support the hypothesis that in middle-income countries education may protect against the obesogenic effects of wealth. We propose that the education gradient may reverse before the wealth gradient. These findings are particularly pertinent to countries where a rapid integration within the global food market may be occurring. In these settings, the combination of a longstanding metabolic and psychological drive for calorie accumulation combined with a sudden influx of heavily marketed high-calorie products, while public health systems are ill-equipped to deal with an emerging non-communicable disease burden, might exacerbate the asymmetry of information between sellers and buyers. This may call for strengthening of global and national governance systems overseeing markets and public health infrastructure alongside promoting access to formal education for women [48]. From this perspective, investment in women's education may be viewed as a public health intervention to address obesity and related diseases benefiting from synergies with multiple other development agendas.
